# Age and stage at diagnosis: a hospital series of 11 women with intellectual disability and breast carcinoma

**DOI:** 10.1186/1471-2407-14-150

**Published:** 2014-03-04

**Authors:** Daniel Satgé, Eric-André Sauleau, William Jacot, Fernand Raffi, Bernard Azéma, Jean-Claude Bouyat, Nicolas El Hage Assaf

**Affiliations:** 1 Epidemiology and Biostatistics department (EA 2415) Oncodéfi project, University Institute for Clinical Research IURC Montpellier 1 University, 641, avenue du Doyen G. Giraud, 34093 Montpellier, France; 2 Biostatistics Department, University of Strasbourg, Faculté de Médecine, 4 rue Kirschleger, 67085 Strasbourg, France; 3 Medical Oncology, Institut Régional du Cancer de Montpellier (ICM), 208 rue des Apothiaires, Cedex 5, 34298 Montpellier, France; 4 Obstetrics and Gynecology, Tulle hospital, Place Maschat, 19012 Tulle, France; 5 CREAI-ORS Languedoc-Roussillon, Q.E. Tournezy 135 allée Sacha Guitry, B.P. 35567 34072 Montpellier, France; 6 19220 Servière le Chateau, France

**Keywords:** Breast cancer, Cancer screening, Cancer stage, Diagnosis delay, Intellectual disability

## Abstract

**Background:**

Breast cancer has been poorly studied in women with intellectual disability (ID), which makes designing a policy for screening the nearly 70 million women with ID in the world difficult. As no data is available in the literature, we evaluated breast cancer at diagnosis in women with ID.

**Methods:**

Women with ID were searched retrospectively among all women treated for invasive breast cancer in a single hospital over 18 years. Age at diagnosis was compared among the whole group of women. Tumor size, lymph node involvement, SBR grade, TNM classification, and AJCC stage were compared to controls matched for age and period of diagnosis using conditional logistic regression.

**Results:**

Among 484 women with invasive breast cancer, 11 had ID. The mean age at diagnosis was 55.6 years in women with ID and 62.4 years in the other women. The mean tumor size in women with ID was 3.53 cm, compared to 1.80 cm in 44 random controls from among the 473 women without ID. Lymph node involvement was observed in 9 of the 11 women with ID compared to 12 of the controls (OR = 11.53, p = 0.002), and metastases were found in 3 of the 11 women with ID compared to 1 of the 44 controls (OR = 12.00, p = 0.031). The AJCC stage was higher in women with ID compared to controls (OR = 3.19, p = 0.010).

**Conclusions:**

Women with ID presented at an earlier age with tumors of a higher AJCC stage than controls despite no significant differences in tumor grade and histological type. Thus, delayed diagnosis may be responsible for the differences between disabled and non-disabled women.

## Background

The life expectancy of people with intellectual disability (ID) has increased, but they are now exposed to greater cancer risk [1]. The global frequency of all cancers has been estimated to be similar in people with ID and the general population [2,3]. Despite differences in cancer distribution according to various organs in persons with ID, breast cancer incidence appears to be similar in women with ID and in non-disabled women [2,4,5]. A lack of research exists in the field of breast cancer in women with ID [6], and very little is known about its clinical presentation, particularly regarding age and tumor stage at diagnosis. However, this information is important for adapted breast cancer monitoring and efficient breast cancer treatment in these women. We present a series of 11 consecutive women with ID treated for invasive breast carcinoma followed in a hospital. The differences we observed from the general population deserve attention, as this question regards nearly 70 million women with ID worldwide.

The frequency of breast cancer in women with ID is not well known. An epidemiological study in Finland reported a similar incidence in the general population [2]. Another epidemiological study in Australia reported a lower incidence of 0.69. However, the authors discussed the possibility that the fewer number of breast cancer cases among women with ID is related to an under-utilization of screening services [5]. An institutional study reported that breast cancer (13 of 51 cases) is the most frequent cancer in women [7]. A Dutch institutional survey reported eight malignancies in women with moderate and severe ID, among which four were breast cancer, suggesting a frequency of breast cancer at least equal to that of the general population [4]. Some conditions, such as Down syndrome [8] and fragile X syndrome [9], occur at a lower frequency in breast cancer patients compared to the general population, whereas other subgroups, such as patients with cerebral palsy [10] or Cowden syndrome [11], occur at a higher frequency among breast cancer patients. For the whole group, including patients with genetic and non-genetic conditions, nulliparity [12], being overweight, and a lack of exercise [6] are factors that increase breast cancer risk.

Although breast cancer is probably the most frequent malignant neoplasm in women with ID, little data is available to support this. The few epidemiological studies [2,3] and institutional reports [4,7] do not provide detailed clinical information. In addition, the few case reports [13-16] do not give a precise idea of the disease for the whole group. Our series of 11 consecutive patients is the first clinically detailed series of women with ID with breast cancer. Our observations of histology, tumor grade, age, tumor size, and disease stage at diagnosis are notable and could have important clinical implications if confirmed.

## Methods

### Patient identification

Ethical approval, as well as permission to create, complete, and access the comprehensive database used in this study, was provided by the Ethics Group of Tulle hospital. Women with ID were searched retrospectively among all women treated for invasive breast cancer (excluding in situ carcinoma) during the 18-year period from 1989 January 1 to 2006 December 31. These dates were chosen because the same pathology team received all breast biopsies and surgical resections during the period. ID is defined as a limitation of intellectual functioning (IQ < 70) associated with a limitation of adaptive behavior first observed before the age of 20 years [13]. This definition excludes patients with late occurring intellectual impairment, such as Alzheimer’s disease. Various psychiatric conditions, such as schizophrenia, were excluded if they were not associated with an ID appearing before the age of 20 years. On the basis of a list of 484 consecutive women with invasive breast cancer, the first step was to ask members of the Gynecology-Obstetrics team of Tulle hospital, particularly physicians, nursing professionals, and secretaries, to recall any patients with intellectual impairment. Hospital records were examined to assess the deficiency and age at onset. In addition, one of us (DS) interviewed the general practitioner (GP) of each of these women directly, searching for intellectual impairment. Identifying ID was easy for women living in an institution for persons with ID and for women followed by the social service involved with ID. For other women, the advice of a psychologist and/or psychiatrist was sought. In a complementary approach, all institutions in the hospital area that shelter patients with ID were contacted by phone, and the psychosocial team, nursing team, and attached GP were interviewed to search for women who developed breast cancer in the institution during the study period. The aim was to determine whether women with ID and breast cancer in the area were treated somewhere other than Tulle hospital. This last search was also to ensure we did not miss patients forgotten by both the hospital team and GPs.

### Patient characteristics

For each patient, we interviewed hospital professionals, GPs, and institution teams, and searched in hospital records and institution records for data on mental state and physical health. Women with ID were classified into three groups: mild ID (IQ 69–50), moderate ID (IQ 49–35), or severe and deep ID (IQ < 34). Data on family diseases, particularly in regards to mental state and cancer, were obtained by interviewing family members when available, and institutional professionals for patients with no known familial link. Regarding cancer, for each woman with ID and breast cancer we noted the age at diagnosis, whether they lived in an institution, the community, or with family, and the mode of tumor discovery. We also noted tumor side, possible multifocality, and the greatest tumor diameter estimated with a microscope. In the case of plurifocal cancer, the diameter was measured for the largest foci. Only the invasive component was measured when in situ carcinoma was associated. The histological type, the Scarff, Bloom, and Richardson (SBR) grade, lymph node involvement, blood metastasis before treatment, and tumor stage were recorded using the TNM classification and AJCC/UICC staging system [17]. Other information regarding treatment and evolution are beyond the scope of this article, which deals with the clinical presentation.

### Comparison with non-disabled women and statistical analysis

The age at diagnosis between women with ID and all non-disabled women in the list of women treated for breast cancer at Tulle hospital was compared by unilateral Student *t* test after assessing normality using a Lilliefors-Kolmogorov-Smirnov test and variance homogeneity using the Bartlett test. Tumor size (greatest diameter), SBR grade, lymph node metastasis, blood metastasis, and stage at diagnosis were compared after matching each patient to controls on the list. Controls were randomly selected after matching for age (in groups of 5 years, e.g., 40–45, 46–50, and 51–55; using the closest year when no case was available within this range) and for the year of diagnosis, or the 2 or 3 years around the year of diagnosis. To maximize the statistical power, four controls were matched with each case. Matching addresses issues of confounding in the study. Without matching, when a sample size is small, the analysis can result in many strata with very sparse data. Little improvement was made in precision by increasing the ratio of controls to cases beyond four [18].

Due to this matching, all analyses were carried out using conditional logistic regression. Statistical tests were considered significant with a 5% alpha risk. Statistical analyses were carried out using the R statistical software [19].

## Results

### Patient characteristics

Among 484 women with breast cancer, three malignant mesenchymal tumors and one lymphoma were excluded, leaving 480 women with breast carcinoma, corresponding to 379 cases of invasive ductal carcinoma, 73 invasive lobular carcinoma, 12 mucinous carcinoma, 10 medullary carcinoma, 3 papillary carcinoma, and one each of fusocellular, spindle cell, and neuroendocrine carcinoma. Ten women with ID were treated for invasive ductal carcinoma and one for mucinous carcinoma. Biological behavior, particularly aggressiveness, varies according to histological type; thus, we limited the comparison of women with ID to non-disabled women with invasive ductal carcinoma.

The etiology of ID was known in three women (Table 1). No genetic condition was identified in the group, including Down syndrome. Five women had mild ID: two with moderate ID and four with severe or deep ID. Four patients were living in an institution. Ten patients were born in the department of Correze, and one arrived from another region of France. Two patients were married. We did not observe that women with ID who developed breast cancer were from families with particular socioeconomic characteristics.

**Table 1:** Patients characteristics in a series of 11 women with intellectual disability and breast carcinoma

ID Level	4 mild ID, 3 moderate ID, 4 severe ID
	4 patients lived in institution, 7 in their family.
ID Etiology	No genetic condition identified, but 1 case of low IQ in the family;
	1 neonatal anoxia, 1 encephalitis at 6 months, 1 stroke at 6 months.
Comorbidities	2 blindness, 2 urinary and fecal incontinence, 2 epilepsy, 2 schizophrenia, 1 mental depression, 1 alcoholism.
Personal and familial cancer	1 patient with sister having breast cancer, 1 patient’s mother with uterine cancer and grand-mother with thyroid cancer, one patient’s father and sister having cancer.
	One patient was treated for a facial basal cell carcinoma

ID=Intellectual disability.

Breast cancer was diagnosed at a mean age of 55.64 years for the 11 women with ID, and 5 out of 11 tumors were found before the age of 50 years. Although no particular action was conducted regarding cancer in women with ID during the study period, tumors were diagnosed at an older age during the first 9 years (mean 65 years) compared to the last 9 years (mean 49 years). Two of the patients discovered the tumor themselves (patients 4 and 7), two tumors were discovered by the GP during a systematic clinical examination (patients 2 and 5). Three tumors were discovered by a family member or a caregiver (patients 3, 6, and 8). Two tumors were found at mammography, one during a screening campaign (patient 9) and the other requested from the GP by the patient (patient 10). In two patients, painful symptomatic bone metastases and pleural metastases revealed the tumor (patients 1 and 11). The tumor characteristics are reported in Table 2. Histological examination of tumors in women with ID did not show particular microscopic features. In Tulle area institutions, we did not find other patients with breast cancer who were treated in another hospital during the study period.

**Table 2:** Breast cancer age and stage at diagnosis in 11 women with intellectual disability

Date of diagnosis	1993-1999 = 5	2000–2006 = 6	
Age at diagnosis	< 50y = 5	50-59y = 2	≥ 60y = 4
Tumor size	≤ 1.9 cm = 2	2–4.9 cm = 6	≥ 5 cm = 3
SBR grade	I = 0	II = 5	III = 6
Lymph node involvement	9/11	1 to 13 nodes involved	
Metastases	3 bones, one associated with pleura, one associated with liver		
TNM classification	T1 N0 M0 = 1	T2 N0 M0 = 1	T2 N1 M0 = 6
	T2 N1 M1 = 1	T3 N1 M1 = 1	T4 N1 M1 = 1

y=years.

### Comparison with non-disabled women

The mean age at diagnosis for the 11 women with ID was 55.64 years (standard deviation [SD] 11.94 years). For all 473 cancers in non-disabled women the mean age at diagnosis was 62.35 years (SD 14.24). The mean age of women with ID was significantly lower than that of the 473 controls (p = 0.047, unilateral Student *t* test). The 11 women with ID were then matched to controls based on age (5-year band), year of diagnosis, and histological type, and the following analyses carried out using conditional logistic regression. The mean tumor size, as evaluated by the mean greatest diameter, was 3.53 cm (range 1.5 – 8 cm, SD 1.87) in the 11 women with ID and 1.80 cm (range 0.3 - 4.5, SD 0.97) in the group of 44 controls. Women with ID exhibited a higher risk of having a greater tumor size than controls (odds ratio [OR] 2.66 [1.26-5.65], p = 0.010 for an increase of 1 cm). Lymph node metastases were found in 9 of the 11 women with ID and 12 of the 44 controls. The OR of a woman with ID having a lymph node was 11.53 [2.40-55.48] (p = 0.002) compared to controls. Visceral metastases at diagnosis were observed in 3 of the 11 women with ID and 1 of the 44 controls (OR 12.00 [1.25-115.4], p = 0.031). Women with ID exhibited a SBR grade of 2 (n = 5) or 3 (n = 6), whereas controls had grades of 1 (n = 6), 2 (n = 18), or 3 (n = 20). When using the grade as a discrete variable, the OR was 1.69 [0.57-5.00] (p = 0.340). The AJCC stage at diagnosis was 1 for 1 woman with ID and 24 controls, 2 for 7 women with ID and 18 controls, 3 for 1 control (and no women with ID), and 4 for 3 women with ID and 1 control (OR using AJCC 1 as the reference category 3.19 [1.32-7.73], p = 0.010, Table 3).

**Table 3:** Regression analysis of breast cancer in 11 women with ID compared to controls

	**OR**	**95% CI**	**p-value**
Tumor size (mm)	2.66	[1.26-5.65]	0.010
Lymph node metastasis	11.53	[2.40-55.48]	0.002
Visceral metastases	12.00	[1.25-115.4]	0.031
SBR grade (reference grade 1)	1.69	[0.57-5.00]	0.340
AJCC stage (reference AJCC 1)	3.19	[1.32-7.73]	0.010

## Discussion

In our series, 11 women with ID accounted for 2.23% of all women treated for invasive breast cancer in the hospital during the 18-year period. As the frequency of ID is estimated to be nearly 2.5% of the population in Western countries [13], this observation is in agreement with the idea that breast cancer is as frequent in women with ID as in women of the general population [2,4,5,7].

### Tumor size and stage

A previous study reported that disabled women tend to be diagnosed with breast cancer at later stages [20]. However, both women with physical disabilities and women with ID were included and, as no information on specific medical conditions was provided, evaluating the extent to which this applies to women with ID in particular is not possible. In our series, women with ID had tumors with greater volume at diagnosis compared to controls, lymph node involvement was more than 11-times more frequent, and blood metastases were 12-times more frequent. Women with ID were more likely to have a higher AJCC stage. The OR was estimated to be 3.2 compared to AJCC 1; thus, women with ID had 3.2-times higher risk of AJCC 2 compared to non–disabled patients. Similarly, women with ID had a 10.2-times higher risk of AJCC 3 (with respect to AJCC 1).

Aggressive breast cancer with a fast growth rate could explain larger tumors and more advanced stage at diagnosis in women with ID compared to non-disabled women. However, because histological type and SBR grade did not significantly differ between the two groups, this possibility is not likely. On the other hand, prolonged delay in the diagnosis of breast cancer has been associated with increased tumor size, increased nodal involvement, and metastasis leading to a more advanced tumor stage [21,22].

### Age at diagnosis

In this series, breast cancer was discovered at the mean age of 55.64 years in women with ID and 62.35 years in the whole group of 473 non-disabled women with breast cancer. The younger age at diagnosis is an unexpected finding of the study. In some genetic conditions associated with ID and increased cancer risk, such as Cowden syndrome, type 1 neurofibromatosis, and Saethre-Chotzen syndrome [11,23,24], breast cancer occurs earlier than in the general population. However, no particular genetic condition was identified in this series. From a practical point of view, particularly for monitoring, whether breast cancer occurs earlier in women with ID needs to be established; if women with ID are diagnosed at an earlier age, different screening guidelines may be needed, such as earlier breast cancer screening for women with ID. In France, the national breast cancer screening policy targets women between 50 and 74 years of age. Applying this policy to the women with ID in our series would have missed nearly half of the tumors because 5 of 11 patients were diagnosed before 50 years of age. In fact, one tumor was discovered in a 63-year-old woman through breast cancer screening, which began in 2001 in the Tulle area. If an earlier age is confirmed by larger samples, research would be needed to understand why women with ID develop breast cancer earlier than non-disabled women.

### Delay in diagnosis

The optimal care of breast cancer relies on early diagnosis. For breast cancer, the policy is based on good general medical follow-up and breast cancer screening in countries where it has been established. Early detection of cancer in individuals with ID is often difficult [25] due to difficulties performing the clinical examination, communication problems, and under-recognized pain by caregivers [26]. In addition, the knowledge regarding breast cancer in women with ID is limited, including signs and symptoms of breast cancer and breast cancer awareness [27,28]. Women with ID do not practice breast self-examination similar to women in the general population and may not fully understand the importance of this surveillance [6,29]. A need exists to train institution staff and women with ID family members in order to enhance our knowledge of breast cancer risk and surveillance [6,30]. The literature on breast cancer screening in women with ID shows that much remains to be done for better monitoring [3,31-34]. Studies in various countries have shown that indications for mammography and uptake of mammography screening are usually lower, and sometimes much lower, in women with ID [31]. Efforts should be made to include more women with ID in screening programs. Previous literature under-evaluated cancer frequency in people with ID [1], and given the lack of clear recent documentation, GPs, families, and caregivers may be less aware of the true cancer risk in women with ID. All of these obstacles and difficulties could explain the delay in breast cancer diagnosis in women with ID. Our study suggests that more attention should be given to an early diagnosis of breast cancer in women with ID.

Our study has some weaknesses: the study is retrospective, few patients were studied (resulting in large confidence intervals, no multivariate analysis was possible), and we did not include population-based data. On the other hand, the strengths of the study are that all patients were followed by the same gynecologic team and tumors evaluated by the same pathology team. The source was the same for patients and controls. In addition, due to the small size of the sample, each patient characteristic could be closely examined. A larger study of breast cancer characteristics at diagnosis is now warranted.

## Conclusion

This first study of breast cancer characteristics at diagnosis in women with ID agrees with recent epidemiological data showing that these cancers are as frequent in women in the general population. We also observed that cancers were found at a more advanced stage and younger age in women with ID than in the general population, despite no significant differences in histological type and tumor grade. These findings warrant a larger study in order to provide a basis for the best care from prevention to long-term treatment of women with ID with breast neoplasia. These findings should encourage work to assess breast cancer risk in women with ID, to search for particular risk factors according to ID level, ID etiology, social level, and screening attendance and to evaluate age at onset for good monitoring.

## Competing interests

All authors have no financial or non-financial competing interests.

## Authors’ contributions

DS worked on the study design, collection of data, data analysis, and manuscript writing. EAS participated in the study design and performed the statistical analysis. WJ was involved in the study design, data interpretation, and manuscript writing. FR performed data collection and assembly and participated in data interpretation. BA participated in data collection, particularly for patients with ID, and in data interpretation. JCB collected patients and participated in data analysis. NEHA was involved in the study design, data analysis, and manuscript writing. All authors read and approved the final manuscript.

## Pre-publication history

The pre-publication history for this paper can be accessed here:

http://www.biomedcentral.com/1471-2407/14/150/prepub
